# Continuous Manufacture and Scale-Up of Theophylline-Nicotinamide Cocrystals

**DOI:** 10.3390/pharmaceutics13030419

**Published:** 2021-03-20

**Authors:** Steven A. Ross, Andrew P. Hurt, Milan Antonijevic, Nicolaos Bouropoulos, Adam Ward, Pat Basford, Mark McAllister, Dennis Douroumis

**Affiliations:** 1 Faculty of Engineering and Science, University of Greenwich, Medway Campus, Chatham Maritime, Kent ME4 4TB, UK; S.A.Ross@greenwich.ac.uk (S.A.R.); A.Hurt@greenwich.ac.uk (A.P.H.); M.Antonijevic@greenwich.ac.uk (M.A.); 2 Department of Materials Science, University of Patras, Rio, 26504 Patras, Greece; nbouro@upatras.gr; 3 Foundation for Research and Technology Hellas, Institute of Chemical Engineering and High Temperature, Chemical Processes, 26504 Patras, Greece; 4 Department of Pharmacy, School of Applied Sciences, University of Huddersfield, Huddersfield, West Yorkshire HD1 3DH, UK; Adam.Ward@pfizer.com; 5 Pfizer Global Research & Development, Ramsgate Road, Sandwich CT13 9NJ, UK; pat.basford@pfizer.com (P.B.); Mark.McAllister@pfizer.com (M.M.)

**Keywords:** cocrystals, hot-melt extrusion, scale-up, continuous manufacturing, X-ray, Rietveld refinement, solubility, surface dissolution imaging, stability

## Abstract

The aim of the study was the manufacturing and scale-up of theophylline-nicotinamide (THL-NIC) pharmaceutical cocrystals processed by hot-melt extrusion (HME). The barrel temperature profile, feed rate and screw speed were found to be the critical processing parameters with a residence time of approximately 47 s for the scaled-up batches. Physicochemical characterization using scanning electron microscopy (SEM), differential scanning calorimetry (DSC), and X-ray diffraction of bulk and extruded materials revealed the formation of high purity cocrystals (98.6%). The quality of THL-NIC remained unchanged under accelerated stability conditions.

## 1. Introduction

Oral drug administration has proven to be the most preferred delivery option in modern medicine with studies showing it to have high patient compliance rates, as well as being more convenient and relatively inexpensive [1]. However, over the past two decades, there has been a substantial increase in the complexity and specificity of drugs. The increased complexity has been accompanied by a decrease in the solubility of the active pharmaceutical ingredient (API) [2]. Approximately 60% of drugs screened in industrial research have poor water solubility, and the traditional formulation of these drugs can lead to poor bioavailability. For a drug to be effective it must be readily available at the target site after administration, bioavailability describes the degree to which a drug can achieve this. After oral delivery, the drug must dissolve in the gastro-intestinal fluid before being absorbed into systemic circulation. Poor solubility can limit drug absorption, thus decreasing its effectiveness. As a result, there has been increased importance in industry and research placed on formulation strategies to enhance the solubility of poorly water-soluble drugs [3].

One such strategy to improve the solubility of poorly soluble APIs is through cocrystallization. Although the exact definition of what constitutes a cocrystal is still being debated this day, a recent perspective authored by 46 specialists defined cocrystals as "solids that are crystalline single-phase materials composed of two or more different molecular and/or ionic compounds generally in a stoichiometric ratio which are neither solvates nor simple salts” [4]. Cocrystallization allows for the modification of components and the chemical and physical properties of a molecule without breaking covalent bonds resulting in a uniquely structured composition [5,6]. The modified interactions will have the potential to improve the physical properties of the API such as the molecules solubility, permeability, particle size, ionization, physical and chemical stability and many more [7,8,9]. This will provide greater bioavailability, whilst the drug molecule itself is unchanged, as all modifications occur at the supramolecular level (intermolecular interactions, hydrogen bonding, molecular packing) [10,11,12]. Studies have found that the multi layered structure of cocrystals improve the mechanical properties, give better flow properties and improve compressibility, which assists the dosage form preparation [13,14].

However, it is also possible to enhance the physical properties of an API by forming salts, solvates, hydrates and polymorphs, to varying degrees of success [15]. It is often the first approach in industry to form a salt after the screening, owing to the fact they are more likely to be physically stable due to the presence of strong ionic bonds [16]. However, in many instances, screening for cocrystals is preferable to screening for salts. For example, Salt formation is not possible when working with neutral molecules, as it requires at least one ionizable point on the API of interest [17]. Furthermore, screening for cocrystals is often preferable when working with weakly basic compounds due to the molecule’s pKa rendering it too low for the salt formation or forming a product with insufficient physical stability to provide an acceptable shelf-life [18]. This is not an obstacle in cocrystallization. In one example stability and solubility of nevirapine, a weakly basic drug was enhanced through cocrystallization with acidic conformers [19]. It is also worth noting that cocrystals can simultaneously address numerous functional groups, while salt formation is directed towards single acidic and basic functional groups. Additionally, cocrystals are not limited to the binary combinations of acid-base pairs as multiple studies have shown the potential of tertiary and quaternary formations [20,21]. Consequently, and despite salt formations preponderance in the pharmaceutical industry, the number of non-toxic and pharmaceutically acceptable acid-base pairs that can be utilized for salt formation is relatively low. In contrast, there is a greater scope of potential API-coformer pairings available in cocrystallization [22]. Salts are also more likely to form hydrates, compared to cocrystals, due to their higher hygroscopicity [23].

It has been shown that cocrystal solubility is directly proportional to the solubility of the constituent reactant and the concentration of the coformer, meaning that through coformer selection it is possible to enhance the physical properties of the API (i.e., melting point, flowability, solubility, permeability, bioavailability, etc...) without hindering their biomechanical action [5]. There are several novel methods to achieve cocrystallization including mechanochemical and liquid assisted grinding, freeze-drying, through slow evaporation of the two components, melt-assisted grinding, slurry methods and a number of emerging approaches using supercritical fluids, microfluids and ultrasound [24]. Despite the growing number of approaches for cocrystal synthesis, full industrial exploitation of cocrystals has thus far been limited in part due to two limiting factors, one or both of which applies to the aforementioned methods: that they are batch controlled and that it is difficult to scale-up production [23,25]. These are challenges that can be overcome through hot-melt extrusion.

Hot-melt extrusion (HME) is one such method of synthesizing cocrystals through temperature controlled, mechanochemical grinding [26,27,28]. HME is a novel technique adopted from the plastic and polymer industries. The HME process involves feeding raw materials through a barrel containing one or more rotary screws towards a die under controlled conditions. The parameters controlled in the HME process include temperature, screw speed, feed rate, residence time and pressure [29,30]. Immense friction takes place between the screw and barrel at high temperatures which provides good mixing of the raw materials, reducing particle size, and breaking down the hydrogen bonds linking the raw materials. New hydrogen bonds will form between complementary pairs during the conveying stage of the HME process and during cooling, forming cocrystals [6,25,27]. Over the last decade, HME has received renewed attention in the pharmaceutical industry as it offers distinct advantages over other commercially available pharmaceutical processing technique [26]. Some of these advantages include the fact it is a solvent-free non-ambient process; is economical offering a reduced production time and having fewer processing steps when compared to other techniques; it is a continuous process; extrudes products in a uniform shape; quality assurance can be easily monitored through process analytical technology (PAT) and that HME has been shown to increases the solubility and bioavailability of insoluble compounds when compared to other processing techniques [30,31,32,33].

One of the other key advantages of HME is that it is relatively simple to scale-up production to an industrial scale [34]. The geometric similarities between mid-size and large scale HMEs enable rapid process scale-up without compromising product quality. Because it is a continuous mechanism the user is easily able to redesign the process to increase throughput and maintain acceptable quality at the same scale. In this study, the effects of the aforementioned processing parameters on the scale-up of pharmaceutical products will be investigated, to evaluate the effectiveness of HME as a tool for industrial cocrystal production. A 1:1 stoichiometric ratio of theophylline (THL), and nicotinamide (NIC) will be utilized as a model API-coformer system, to investigate the effect of these parameters in the scale-up of production via HME. These two materials have been selected because THL-NIC cocrystals have been used in a wide array of publications and there is sufficient information available on THL-NIC crystal structure to reliably use as a model drug to evaluate the purity of our product produced under different conditions [32,33,34,35]. The main aim of the study is to provide a scale-up paradigm of high-quality cocrystals processed by HME. By adjusting critical process parameters (CPP) we were able to manufacture pharmaceutical cocrystals with high throughput in a continuous manner.

## 2. Materials and Methods

### 2.1. Materials

Theophylline anhydrous, 99%, powder and Nicotinamide 98% (TLC), powder were purchased from Sigma Aldrich (Gillingham, UK) and used without any further treatment. All solvents used for HPLC were analytical grade.

### 2.2. Hot Melt Extrusion Continuous Manufacturing and Feed Rate Calibration

Extrusion was performed on a co-rotating twin-screw extruder (Eurolab-16, Thermo Fisher, Germany), at 100 rpm. The maximum extrusion temperature can be seen in Table 1, and a breakdown of temperature parameters for each individual heating zone can be found in the Supplementary Materials. The feed rate was set at 76% to produce 1.5 kg/h of THL-NIC cocrystals. A mixture of THL and NIC were blended to produce a uniform batch at a 1:1 molar ratio in a Turbula TF2 mixer (100 rpm) (Basel, Switzerland) for 10 min. The physical mixture was then added to the feeder. The volumetric powder feeder (Brabender Duisburg, Germany) was calibrated to provide a throughput of 500 g/h and 1.5 kg/h (data not shown). From these studies, it was found that a feed rate of 25% would throughput 500 g/h and at 76% the feeder will throughput 1.5 kg/h. The screw speeds varied from 70–100 rpm. An extrusion die was not used during the processing. It has been shown that cocrystal conversion during extrusion processing is more likely when using a screw design comprising of a mixture of forward conveying, forward mixing (30°/60° screw angles) and neutral mixing (0°/90°) elements [36]. Such a design allows for both dispersive and distributive mixing. As such, a similar screw design as proposed by Dhumal et al. will be utilized in this study. The screw design comprises 4 conveying zones, 3 mixing zones and a discharge zone. The material is fed into the first conveying zone and discharged from the HME from the discharge screw elements at the end of the final conveying zone. Sandwiched between each different conveying zone are 3 separate kneading zones. The first kneading zone comprises 10 screw elements in a forwarding mixing design at 30°, 60° and 90° angles. The second kneading zone 6 neutral mixing elements at 0° and 90° angles. This high shear kneading zone was added to the screw design to induce additional kinetic energy to break down the stronger theophylline hydrogen bonds [37]. The third kneading zone used the same configuration and number of elements as the first. An image of the screw configuration can be found in the Supplementary Materials.

### 2.3. Residence Time

**Figure 1:**
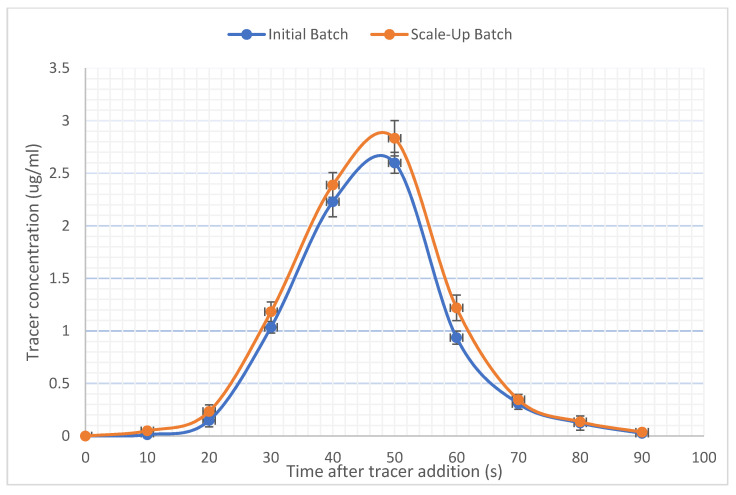
The graph shows the residence time distribution (RTD) of the initial (F3) and scaled-up (F6) batches derived by using the following equations [44,45].

Residence time distribution was ascertained for the cocrystals by following the same method as described in 3.3, but with the addition of a tracer to add colouration to the processed material. Residence time studies were only carried out on the optimized processing settings for the initial and scale-up batches. In this instance, the extrusion settings used for this study were F3 and F4 (Figure 1). The extruder was left running for 5 min to ensure the operation was stable, with the torque averaging at 57%. After 5 min 30 mg of sodium fluorescein salt (Sigma-Aldrich, Dorset, UK) was added as a tracer into the extruder. Samples were taken at the extruder die every 10 s for 90 s. Quantification of sodium fluorescein was performed by taking 5mg of powder from each sample and dissolving it in 20 mL of water. The samples were then placed in a UV spectrophotometer (Jenway 6305, Bibby Scientific, Chelmsford, UK) for analysis at 475 nm, where fluorescein exhibits maximum absorption. The sodium fluorescein could easily be visually differentiated from the other extrudates, due to its dark orange colour. All absorbance values obtained from the coloured extrudates were used to plot a graph of absorbance vs. time to determine the residence time distribution (*n* = 3).

### 2.4. Scanning Electron Microscopy

Scanning Electron Microscopy (SEM) was used to examine the morphological features of the bulk materials, physical mixtures and cocrystals. The samples were mounted on aluminium stubs and gold-sputtered, using an Edwards S150B sputter coater (Edwards Vacuum, Burgess Hill, UK) under an argon atmosphere at a pressure of 7 mbar for 1 min. The SEM measurements were carried out on a Zeiss EVO MA10 scanning electron microscope (Carl Zeiss AG, Jena, Germany), with the accelerating voltage of the electron beam set at 11 kV.

### 2.5. Particle Size Distribution

Laser diffraction was used to measure the particle size distribution of the THL, NIC and THL-NIC cocrystals. This was achieved using a dry powder dispersion unit (Scirocco 2000) of a Mastersizer 2000 laser diffraction particle size analyzer (Malvern, Worcestershire, Malvern UK). 5 g of each sample was placed in a vibratory tray, which fed the powder into the sample dispersion unit for sizing. Sampling time was set at 15 s, and each sample was measured in triplicate.

### 2.6. Differential Scanning Calorimetry (DSC)

The temperature profiles of the bulk THL and NIC, the THL-NIC physical mixture, and the extruded THL-NIC cocrystals produced under a variety of different parameters were analysed using a differential scanning calorimeter (Mettler Toledo 823e, Greifensee, Switzerland). The samples were accurately weighed between 3 mg–5 mg and placed into an aluminium pan and crimped. With the exception of the bulk nicotinamide, which was heated to 140 °C, each sample was heated from 25 °C to 280 °C at a scan rate of 10 °C/min with a gas flow of 50 mL/min. The nitrogen gas flow rate was set at 50 mL/min. STARe excellence software was used to analyse the data. The DSC instrument was pre-calibrated using indium and zinc as standards with regards to both temperature and enthalpy.

### 2.7. X-ray Powder Diffraction

XRPD data were collected using a D8 advanced X-ray Diffractometer (Bruker, Germany) in theta-theta geometry using the reflection mode. A Cu anode X-ray tube was powered at 40 kV and 40 mA. A primary Göbel mirror was used for the parallel beam and the removal of Cu Kβ. A primary 4o Soller slit and a secondary 2.5o Soller slit, a 0.2 mm exit slit were selected for this experiment. Data was collected between 2-40o 2θ with a step size set at 02o 2θ and the counting time set at 0.5 s per step. The detector contains 176 active channels, so the total counting time is 52.8 s per step. The sample rotation was set at 15 rpm. EVA phase analysis software (Bruker, Germany) [38,39] was used to identify peak positions and intensities of the bulk and extruded products. To verify whether we had successfully produced cocrystals, we retrieved the crystal structures for THL-NIC cocrystals from the Cambridge crystal database (CSD) and performed Rietveld refinements using the TOPAS V4.2 program (Bruker). The CSD refcodes for THL, NIC and THL-NIC cocrystal are BAPLOT01, NICOAM02, UNEZES respectively. The crystal structure data taken from the CSD was fitted to the diffraction peaks from our cocrystals, to see how closely the two matched. Standard peaks, taken from the CSD, for the bulk products were also fitted to our structure to identify peaks of any remaining THL or NIC. This enabled us to find what percentage of our batch underwent cocrystallization.

### 2.8. In Vitro Dissolution Study

The dissolution studies were conducted on a Varian 705 DS dissolution paddle apparatus (Agilent Technologies, Inc., Cary, NC, USA). In this study 1000 mg of the extruded cocrystals and physical mixture and were placed in 900 mL of water at 37 1 °C, in line with standard US Pharmacopeia methodology. A standard solution having a known concentration of theophylline in the same medium and under the same conditions. The paddles stirred the solution for 2 h at 50 rpm to dissolve the powders. The samples were collected and then filtered at predetermined time intervals.

### 2.9. HPLC Analysis

The amount of THL present was determined with the use of HPLC analysis. An Agilent Technology 1200 series system (Agilent Technologies, Cheadle, UK) equipped with a hypersil-5ODS, 100 mm◦ø 4.6 mm ID column was used for the HPLC assay. The mobile phase consisted of, methanol, water (15:85, *v*/*v*), with a 2.1 mL/min 1 flow rate. The eluent was monitored with a UV detector at 254 nm, the injection volume was set at 20 µm and the run time was 5 min. The results were integrated using Chemstation software. The retention time of the THL was 3.1 min. A THL calibration curve, at concentrations varying from 10 g/mL to 100 g/mL, was constructed and used to evaluate the samples.

### 2.10. Surface Dissolution Imaging

Surface dissolution imaging (SDI) was utilized to investigate dissolution behaviour and establish the intrinsic dissolution rate (IDR) for both the bulk materials and extruded cocrystals. SDI was carried out using a Sirius SDI 300 (Forest Row, UK) fitted with ActiPix UV area-imaging technology. This apparatus comprises a flow cell and sample holder, an integrated syringe pump, temperature control unit, UV lamp and detector and bespoke data analysis software. The main purpose of the syringe pump is to feed the dis- solution medium through the temperature control unit, into a flow cell where the dissolution process occurs. The flow cell consists of quartz and a sample holder. Once the compact holder has been placed into the sample holder and inserted into the quartz cell, where the UV light source of the SDI 300 UV imager is a pulsed Xenon lamp with a replaceable wavelength filter allowing high-quality UV images to be taken. Here the dissolution experiments were performed in 200 mL of 6.5 pH phosphate buffer, under a flow rate of 0.2 mL/min, for 20 min. The UV detector was set at 215 nm and the temperature was set to 37 °C. Each sample was run in triplicate. Approximately 5mg of each powder was placed in a stainless steel sample cup, which was then compressed for 1 min, using a quickset minor torque wrench (Torqueleader, M.H.H. engineering Co. Ltd., Guildford, UK), set at a constant pressure of 40 cN.m. IDR values were calculated using calculated extinction coefficients for each of the compounds.

### 2.11. Stability Studies

The extruded samples and physical mixture were placed in a sealed desiccator under accelerated conditions of 40 ± 1 °C and 75 ± 1.5% RH as per ICH guidelines, for a period of two months to ascertain the stability of the cocrystals at extreme accelerated conditions [40]. The samples held at elevated conditions were then characterized through XRPD analysis (the same method used as in 3.7), to investigate any loss in crystallinity.

## 3. Results

### 3.1. Hot Melt Extrusion Continuous Processing

To identify the optimum processing parameters during the HME process a twin-screw extruder was used to process a 1:1 molar ratio of THL/NIC. The two most common variables during scale-up are barrel temperature and screw speed. Typically, as the batch size is increased the temperature must also increase. This is done to allow the increased product between the screws and the barrel wall to absorb the heat. If the temperature is not increased a percentage of the product may not be sufficiently heated and cocrystals will not form, resulting in a batch of poor purity [6]. This was shown by Moyadiya et al. when scaling-up the production of Indomethacin-saccharin cocrystals, who found that when increasing a batch size of 0.1 and 0.3 kg/h to 1 kg/h, that the temperature of the process requires adjustment to maintain cocrystal purity [25]. When scaling-up the batch produced at 0.1 kg/h 3-fold to 0.3 kg/h, the maximum barrel temperature had to be raised from 155 °C to 165 °C. Then, when further scaling from 0.3 kg/h to 1 kg/h, the barrel temperature again had to be raised by 10 °C to 175 °C, to allow for full cocrystal conversion. In another study, Dhumal et al. emphasized the importance of screw configuration in regards to extrusion processing, though once a suitable configuration was established, did not find it to be a significant factor in the scale-up of production [36]. Screw speed must also be increased with the feed rate, otherwise, the extruder will clog [41,42].

Initially, the feeder was calibrated to extrude at 0.5 kg/h to scale up to 1.5 kg/h once the optimal conditions were discovered. The physical mixture was extruded at three different temperatures (145, 165 and 185 °C) to find the optimal temperature for extrusion. Based on the solid-state analysis (see below), it was found that extrusion at 145 °C did not fully convert the physical mixture to cocrystals, with large portions of THL and NIC remaining in the extrudates. It was also found that THL would begin to decompose when processed at 185 °C while at 165 °C, with a screw speed of 100 rpm would provide a more stable and highly crystallized batch than the other temperatures, but still contained notable amounts of pure THL and NIC. When the screw speed was reduced to 70 rpm with the temperature kept at 165 °C, the resulting batch displayed stable and high crystallinity cocrystals. This is likely due to the fact the physical blend would have spent longer in the extruder allowing it to become sufficiently heated. However, when the process was scaled-up to 1.5 kg/h, the twin-screw extruder reached maximum torque, causing the process to fail. The screw speed was adjusted to 100 rpm and under increased throughput produced a highly crystalline and stable batch.

These findings are in disagreement with previously published work from Moradiya et al. as this work did not find the temperature to be a significant factor in the scale-up of the extrusion process [25]. Any change in temperature to this extruded batch caused either a partially cocrystallized patch, giving pure cocrystal purity or induced thermal degradation of the extruded product. Instead, the scale-up technique was controlled purely by adjusting the screw speed to accommodate the change in feed rate. Here, as the feed rate increased, the screw speed was steadily risen, with the discharge material collected and characterized. Through increasing the screw speed by 15 rpm per trial after scaling the method from 0.5 kg/h to 1.5 kg/h, it was eventually found after two trials that an increase of 30 rpm allowed for the production of pure cocrystals. The complete table of experiments is shown in Table 1.

### 3.2. Residence Time

The residence time describes the amount of time that a particle will spend in the extruder and to the extent, it will participate in the extrusion process, as such, it will highly affect the quality of the product [43]. A low residence time could possibly lead to inadequate dispersion of particles and could cause the product to not be sufficiently heated, leading to an impure extrudate. If the cocrystal constituents are passed through the extrusion barrel too quickly, then they will not be exposed to the excess energy present in the HME process for long enough to induce the eutectic melting necessary to form cocrystals. This will lead to a partially cocrystallized batch [35,39,42] Whereas a high residence time could lead to thermal decomposition, due to the cocrystal constituents being held in conditions of high thermal and kinetic energy for too long. [31,44]. Therefore, it was important to investigate the residence time’s effect on formulations as well as other parameters in the extrusion process. This was investigated for the initial batch (F3) and the Scaled-up batch (F6) and can be seen in Figure 1. The residence time distribution was found to be 46.7 s with a time delay of 20 s and then an increase to reach a maximum within 40–50 s followed by a relatively rapid decrease [45,46]. As expected, the residence time distribution reduced with an increase in the screw speed. The mean residence time was estimated at 48.1 s for F3 and 48.0 s for F6. These values are derived by using the following equations [47,48]:

(1) $$E\left(t\right)=\frac{C\left(t\right)}{\sum^{\;}C\left(t\right)\Delta t}$$

(2) $$t=\frac{\sum^{\;}tC\left(t\right)\Delta t}{\sum^{\;}C\left(t\right)\Delta t}$$

where *C*(*t*) is the tracer concentration, ∆*t* is the sampling period and t the sampling time [45].

### 3.3. Scanning Electron Microscopy

As can be seen from Figure 1, the residence times for both F3 and F6 are near identical despite the differences in processing parameters between them. With no change in temperature or screw configuration, the increase in throughput has been accommodated by adjusting the screw speed alone, allowing for a near-identical residence time between the two extrusion settings. In comparing this work to previously published work from Moyadiya et al., who did not thoroughly investigate the effect of residence times during scale-up, this work has found that in maintaining a similar residence time between the initial and scaled-up batches, it is possible to produce the same high-quality cocrystals [25]. By increasing the screw speed by 30 rpm between the initial and scaled-up batches, it was possible to maintain the same residence time and cocrystal purity, without the alteration of temperature.

**Figure 2:**
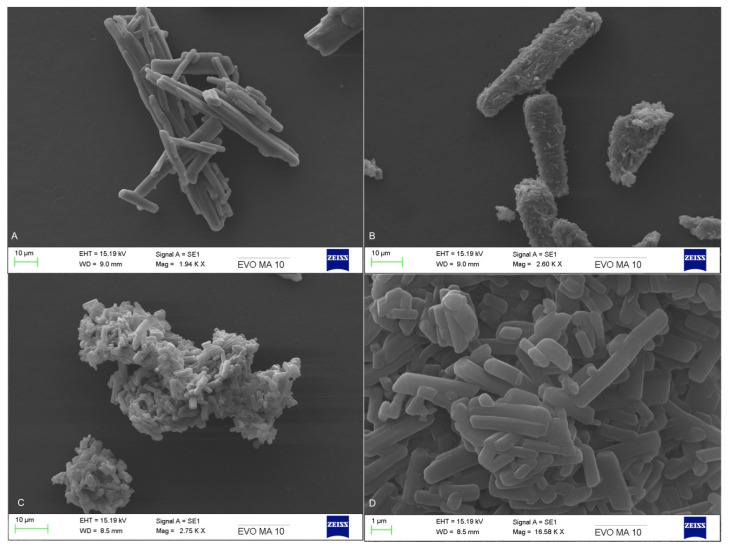
SEM analysis of (**a**) bulk THL, (**b**) THL-NIC physical mixture, (**c**) THL-NIC cocrystals at 2.75 kX magnification, (**d**) THL-NIC cocrystals at 16. 58 kX magnification.

SEM analysis revealed significant morphological differences between the bulk materials and the extruded cocrystals. As it can be seen in Figure 2, bulk THL particles appear elongated with a tubular and acicular shape while the THL-NIC physical mixture as irregular shaped particles. Figure 2c,d images depict the shape of extruded cocrystals (pre and post-scale-up) which show the formation of agglomerates with the fractured network—like surface morphology [49]. The particle morphology of all extruded batches was identical suggesting the manufacturing of extruded cocrystals in a reproducible manner.

### 3.4. Particle Size Analysis

**Figure 3:**
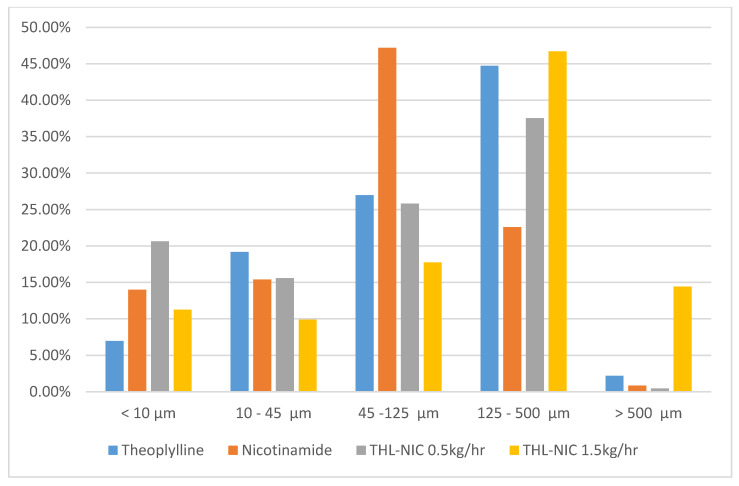
Graph showing the particle size distribution for the bulk products and extruded cocrystals.

Particle size analysis shows (Figure 3) similar particle size distribution between the bulk THL and the cocrystals, with the majority between 125 µm and 500 µm. Interestingly, the cocrystal batch extruded at 1.5 kg/h had a larger particle size distribution than the cocrystals extruded at 0.5 kg/h, with just under 15% of the batch larger than 500 µm. In contrast, less than 1% of the cocrystals extruded at 0.5 kg/h were larger than 500 µm. This may be explained by the fact that the cocrystals produced after the scale-up were extruded at 100 rpm, facilitating greater high shear kneading in the mixing zones, causing greater interaction between the particles producing greater kinetic energy in the extruder barrel. This will lead to a more effective deagglomeration, as the particles come into close contact at high speeds, preventing aggregates from forming during the grinding process [12].

### 3.5. Thermal Analysis

**Figure 4:**
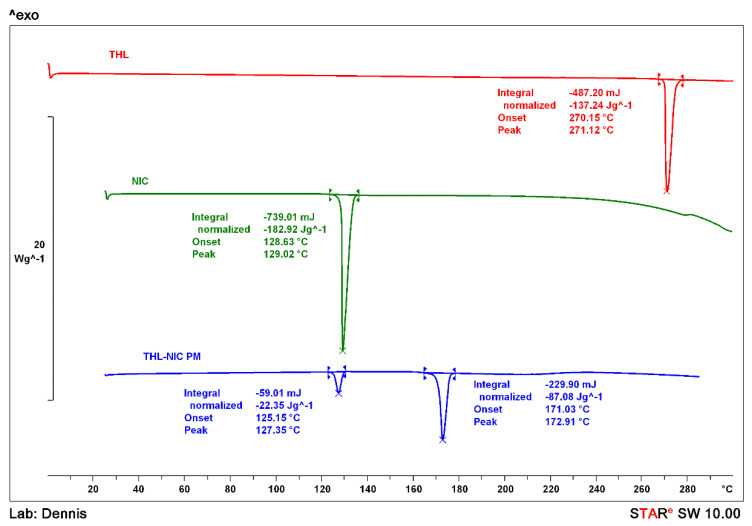
Differential scanning calorimetry (DSC) thermograms of bulk THL, bulk NIC and the THL-NIC physical mixture.

Bulk THL, NIC and the physical mixture were analysed via DSC so the samples could be easily differentiated based upon the endothermic events. As shown in Figure 4, THL and NIC presented melting endotherms with an onset of 270.15 °C and 128.63 °C, respectively [50,51]. The thermogram for the THL-NIC physical mixture shows two clear endothermic peaks, the first of which appears just below the melting point of NIC whilst the second appears at 171.03 °C. This peak is can be attributed to the melting point of THL-NIC cocrystals, as reported in the literature, suggesting that cocrystallization may have occurred on heating the physical mixture [51]. The first endothermic peak is associated with the eutectic melting, followed immediately after by a slight exothermic event at ~132 °C, indicating the cocrystallization of the two components. Another endothermic point will then be observed at the cocrystals melting point. This effect commonly occurs in complementary API-Coformer pairs when heated together, and is often employed as a mechanism of cocrystal screening. In situations where this effect is not seen, it is likely no H-bond interactions are taking place between the two components, meaning cocrystal formation is not possible between them [6,52].

**Figure 5:**
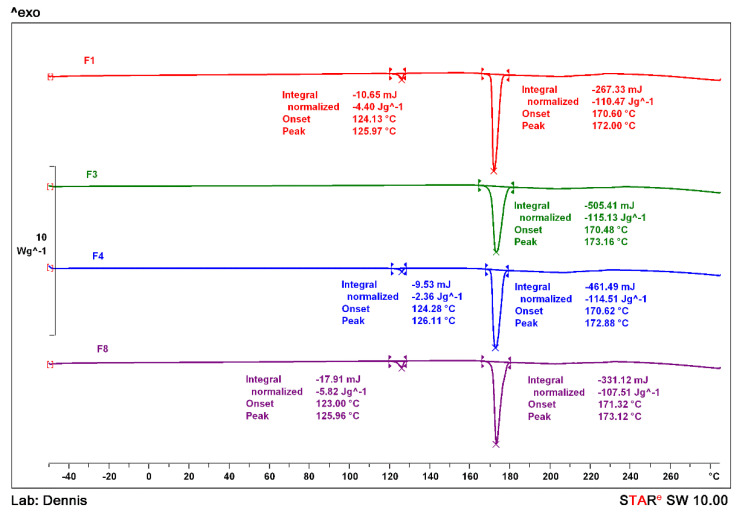
DSC thermogram of THL-NIC extrudates processed at various extrusion settings.

To identify the optimal parameters to extrude the cocrystals, the THL/NIC physical mixtures were extruded at a number of temperatures and screw speeds as shown in Table 1 The product from each was analysed F3 conditions displayed a single melting point at 170.48 °C (Figure 5), suggesting that the conditions under which this formulation was extruded, are the optimal setting for cocrystal formation at the defined throughput.

The single peak indicates that no other substance is present in the sample, as if bulk, NIC were present, there would be another peak at ~ 127 °C. The THL-NIC cocrystals melting point at 1720.48 °C is near identical to examples in literature [51] By contrast, F1 displays a peak at 124.13 °C suggesting traces of NIC remain in this batch, thus the bulk materials did not fully undergo cocrystallization. This likely occurred because the extrudate was not sufficiently heated or time spent in the extruder was not long enough to convert a number of individual crystals into cocrystals.

The melting point of NIC has shifted slightly to a lower temperature compared to the pure NIC sample. This is often observed in physical mixtures where compounds of the mixture act as impurities to each other causing depression in a melting point. Similar results can be observed with F8, where a peak is also present at 123 °C, suggesting the presence of pure NIC. It is also interesting to note that the melting point for the cocrystals is slightly broader than batches prepared at lower temperatures. This suggests the cocrystals are beginning to physically degrade (deformation of cocrystal) under the higher HME processing temperature. This could be explained by the higher energy level of the system under consideration, hence NIC and THL had the opportunity to create crystals even though they are not thermodynamically favourable [52,53]. A similar peak at 124.28 °C can be seen for F4, but there is less enthalpy given off for this formulation (−2.36 Jg^−1^) than there is in F1 and F8. This indicates that less NIC is present in this sample. This is likely because F4 was extruded at the optimal temperature but the higher screw speed of 85 rpm [52]. This change would have led to a decrease in residence time, as the individual components would spend less time under the temperature, meaning that not enough heat was applied to fully convert the entire batch.

**Figure 6:**
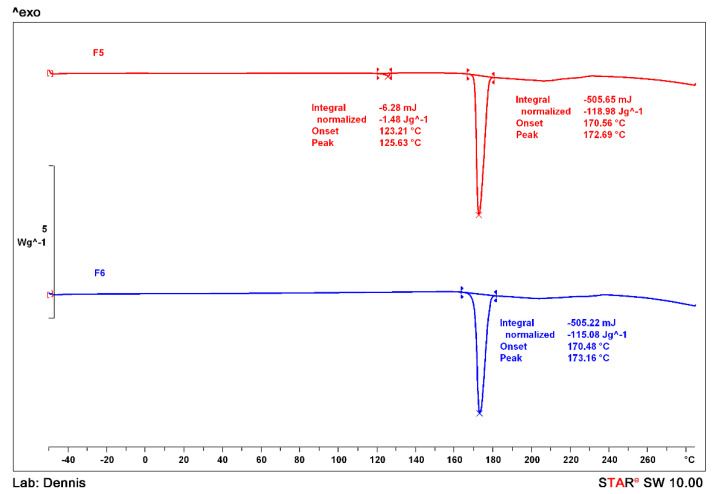
DSC thermogram of THL-NIC cocrystals after scaling up to 1.5 kg/h.

After establishing the optimal extrusion parameters at 0.5 kg/h, production was scaled up to 1.5 kg/h. However, using the same extrusion parameters, as used in F3 did not scale; the extruder became clogged due to the increased amount of material added, to compensate, the screw speed was raised to 100 rpm. Although extruding at this screw speed caused impurities when producing lower amounts, at 1.5 kg/h the increased amount led to a longer residence time, ensuring that the crystals spend a long enough time in the extruder. The thermogram (Figure 6) showed a single melting point at 170.48 for F6, which is identical to the scaled down F3, suggesting the batch has fully undergone cocrystallization [53]. This demonstrates that the scale up of THL-NIC cocrystals is easily possible by only editing a single processing parameter (screw speed).

### 3.6. X-ray Powder Diffraction

**Figure 7:**
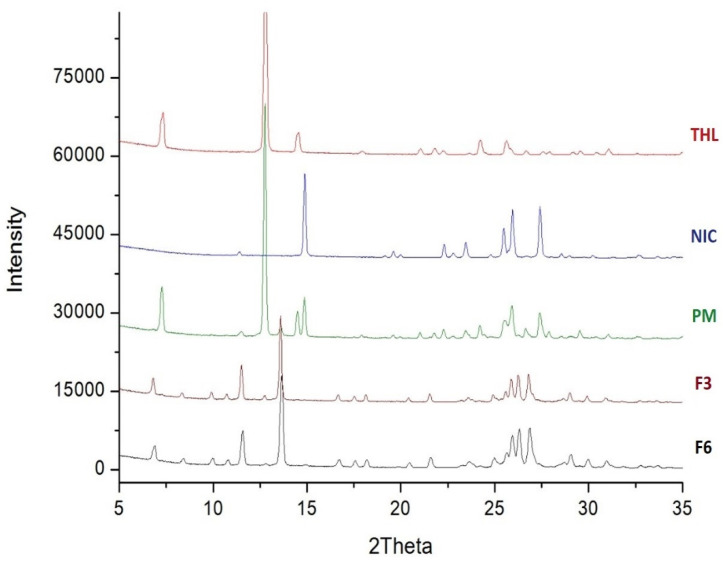
Diffractogram showing the peak positions (2θ) and intensities and of THL, NIC, the THL-NIC physical mixture (PM), F3 (THL-NIC at 0.5 kg/h, 165 °C, 70 rpm), F6 (THL-NIC at 1.5 kg/h, 165 °C, 100 rpm).

Samples were further analysed by XRPD to identify the diffraction patterns. These we then compared to documented standards taken from the Cambridge Structural Database (CSD). The formation of 1:1 THL-NIC cocrystals was initially reported by Lu and Rohani, which has since been replicated by others [51]. As shown in Figure 7, the main intensity peaks for THL appear at 7.3°, 12.5°, 14.7°, 21.5°, 22°, 22.3°, 24.4° and 25.9° 2θ while those of pure NIC appears at 11.3°, 14.9°, 19.6°, 22.4°, 23.5°, 25.6°, 26° and 27.5° 2θ values, respectively. In contrast, the main diffraction peaks of the THL- NIC cocrystals (both before and after scale-up) appear at 7.1°, 8.3°, 10°, 10.9°, 11.8°, 13.5°, 17°, 17.5°, 17.9°, 20.4° and 21.9° 2θ values, as well as a cluster of peaks seen between 25◦ and 27.5°2θ. The XRPD pattern for the THL-NIC sample prepared in this study is completely different from the individual bulk substances and comply with the reference, which indicates the successful synthesis of the cocrystal [32,45,46]. The peak locations for the cocrystals extruded at 0.5 kg/h and 1.5 kg/h are almost entirely similar indicating that the scale-up caused no major difference in the crystalline structure. The peak intensities for the sample extruded at 1.5 kg/h are slightly higher than the samples extruded at 0.5 kg/h indicating a slightly higher percentage of crystallinity, though this is the difference is small and not significant [54,55].

**Figure 8:**
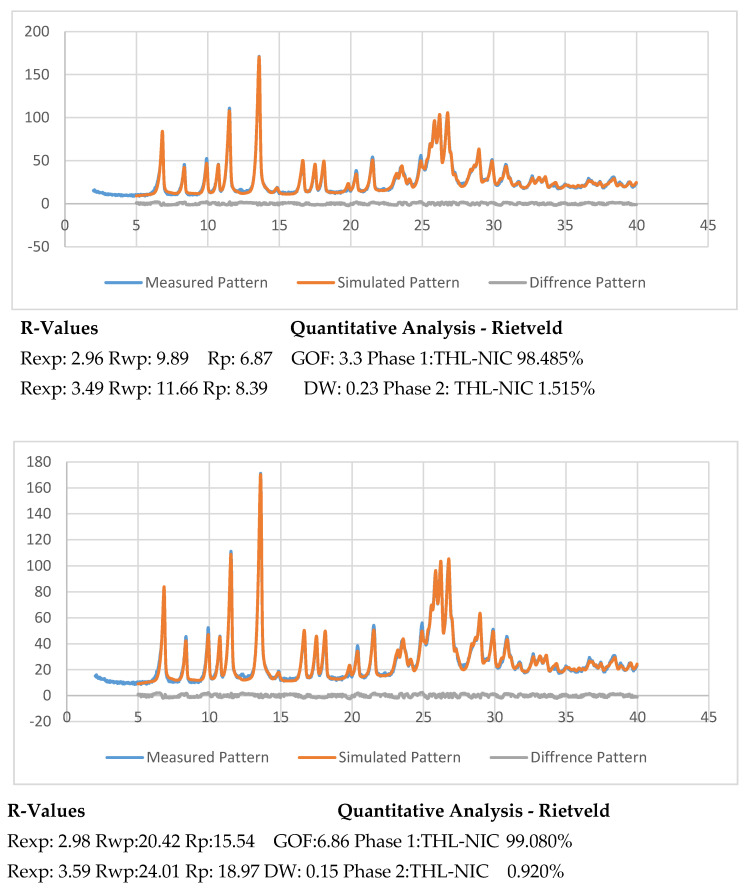
Rietveld refinement of the X-ray powder diffraction (XRPD) data for THL-NIC cocrystals extruded at 500 g/h (**Top**) and 1.5 kg/h (**Bottom**), after 12 months at accelerated conditions where the measured pattern is represented with the blue line, the simulated pattern with the red line and the different pattern in grey. Refinement values displayed below.

The percentage of the batch which successfully underwent cocrystallization was determined from the XRPD data by employing Rietveld refinement with favourable residual variances. This was achieved using TOPAS V4.2 (Bruker). The results from this experiment were then compared to the simulated results obtained from the CSD [56]. Though what constitutes a good fitting with R-factors in Rietveld analysis is not currently clear, it is generally agreed that if weighted profile R-factor (Rwp) is within or around 3x the expected R factor (Rexp) the result is good [57]. The best fit between the simulated and experimental powder patterns was obtained with Rwp = 9.89, Rexp = 2.96 for the sample extruded at 0.5 kg/h and Rwp = 20.42, Rexp = 2.98 for the sample extruded at 1.5 kg/h. The Rwp for this experiment is just outside this range, so for the purposes of this experiment, the fit is acceptable. The extruded cocrystals were analysed alongside a simulated pattern [32] to find the difference between the two. The samples produced at 0.5 kg/h composed of 98.48% cocrystal, while the batch produced at 1.5 kg/h contained 98.57% cocrystal (Figure 8). To identify the remaining content of the sample we fitted the CSD structure for pure THL and NIC to our cocrystals. This revealed the remaining amount to be uncrystallized Nicotinamide, however, the amount is minuscule at 1.5% and even less for the samples produced at 1.5 kg/hr at 0.9% [58,59]. From the results of this experiment, it can be concluded that the scale-up process had an insignificant effect on the purity of the cocrystals.

### 3.7. In-Vitro Dissolution

One of the major advantages of cocrystals is there improved solubility over the bulk products and as such it is one of the key criteria to assess their performance. However, THL is a BCS Class I drug, meaning it is already highly soluble. In this study, theophylline is being used as a model drug to assess the scalability of the HME process, so while improving the dissolution/bioavailability is not the aim it is important to assess whether the scale-up process affects the solubility of the drug. If the scale-up process has a significant, negative impact on the solubility, it would render the process obsolete. For this reason, the dissolution patterns of the cocrystals before and after scale-up were compared to that of the bulk THL. The particle size of the cocrystals has been shown to influence the dissolution rates. Slower dissolution rates have been observed for particles larger than 500 µm in cocrystals [60]. Because of this, the particle size distribution was also measured for the bulk THL, bulk NIC, and the extruded cocrystals to assist with the analysis of the dissolution rates.

**Figure 9:**
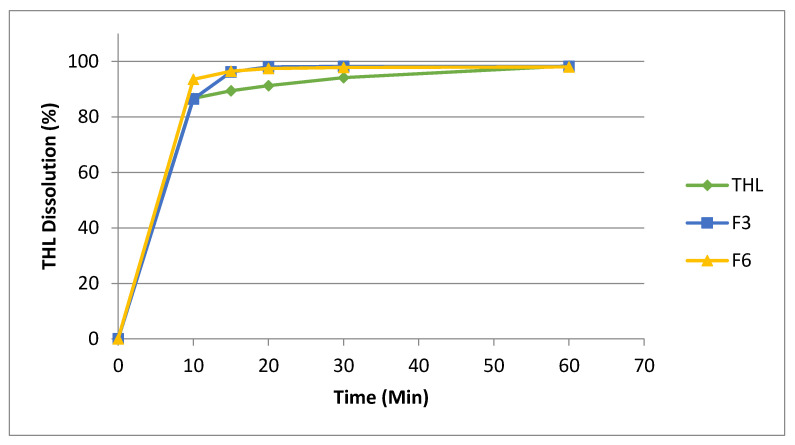
Dissolution profiles bulk THL, THL-NIC cocrystals extruded at 0.5 kg/h (F3) and cocrystals extruded at 1.5 kg/h (F6).

As shown in Figure 9, the bulk THL had a rapid dissolution rate with approximately 86.7% of the drug is fully dissolved in 10 min and over 98.7% of the bulk THL had fully dissolved in 60 min. The cocrystal extrudates demonstrated similar dissolution rates to the bulk THL, with dissolution rates varying from 98.2–99.2% within 60 min before and after the cocrystal scale-processing. This data is supported by the particle size distribution (Figure 3). It should be noted, however, that the higher percentage of larger particles had little effect on the solubility. The THL/NIC cocrystals were found to have a quicker dissolution rate than the bulk THL. It can be seen that the dissolution profile for both cocrystals plateau just after 20 min, releasing over 98% of THL in this short space of time. In contrast, only 90% of bulk THL had successfully dissolved by 20 min, with it not reaching a plateau before 60 min. This is likely due to the presence of the NIC coformer, with the more readily soluble NIC, assisting dissolution [61]. From this, it can be concluded that neither the cocrystallization process nor the scaling-up process had an adverse effect on the solubility of the theophylline [59].

### 3.8. Surface Dissolution Imaging

**Figure 10:**
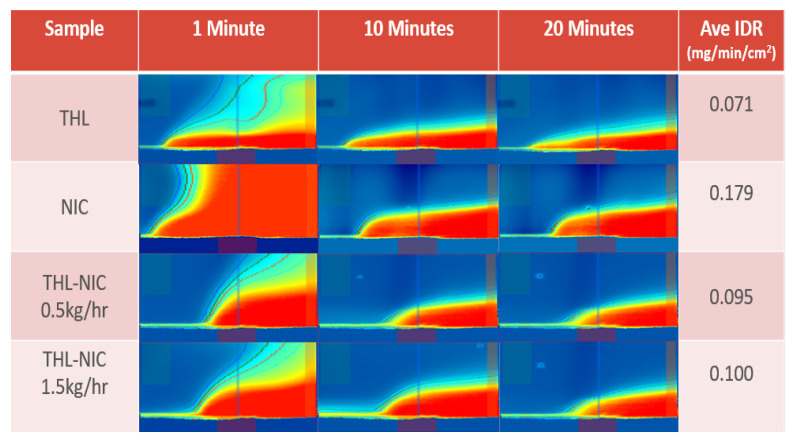
Graph showing the average intrinsic dissolution rate and UV images of bulk substances and extruded THL-NIC cocrystals surface dissolution after 1, 10 and 20 min.

The surface dissolution profiles were visually observed, and the intrinsic dissolution rate (IDR) calculated via surface dissolution imaging. The samples extinction coefficients were calculated via UV spectrophotometry, revealing absorbance to be highest at 214 nm, so the UV detector was set as close as possible to this wavelength. As can be seen from Figure 10, All compounds were soluble in pH 6.5 Phosphate Buffer, as expected showing the extrusion process did not negatively affect the cocrystals solubility [62]. This study supports the HPLC data indicating that cocrystallization alongside the far more soluble NIC has improved the dissolution rate of the cocrystals, with the average IDR for the 0.5 kg/h and 1.5 kg/h cocrystals being 0.095 and 0.1 respectively, in contrast to bulk THL’s IDR value of 0.071 [60,63,64,65,66]. Owing to its kinetic nature, IDR assumes a better correlation with in vivo drug dissolution dynamics than solubility, so it can be confidently stated, that the cocrystallization process has increased the solubility of the THL [64]. Furthermore, with both the initial and scaled-up batches of cocrystals displaying a similar IDR and flow profile over 20 min it can be stated that the scale-up process has not significantly affected the solubility properties of the cocrystals [62].

### 3.9. Stability Studies

**Figure 11:**
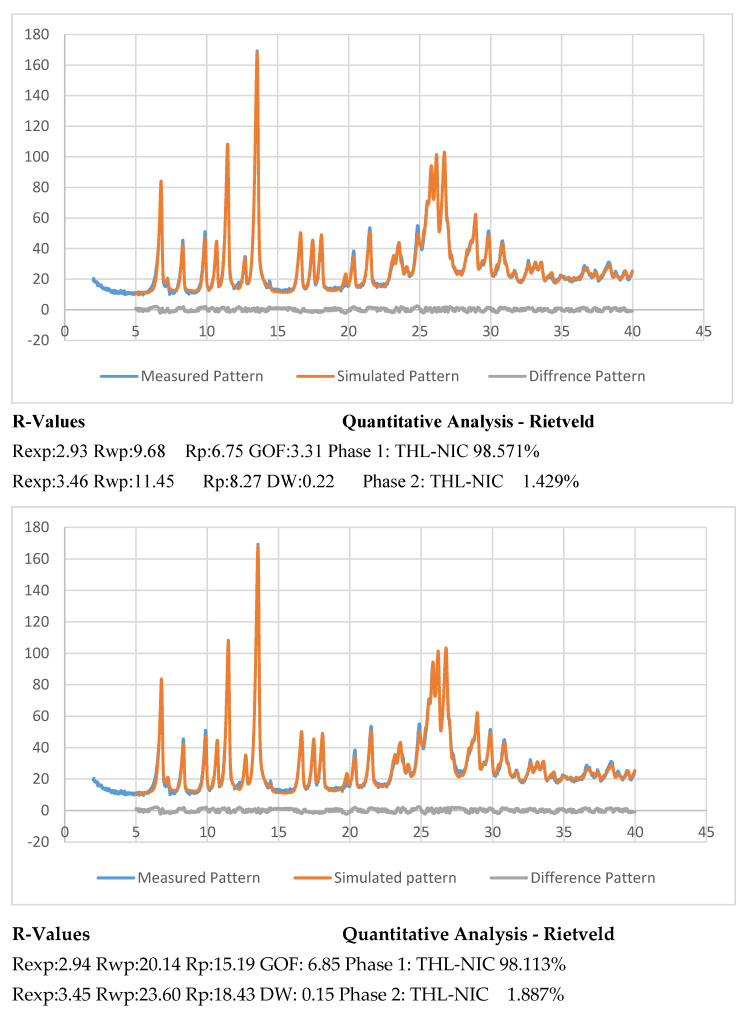
Rietveld refinement of the XRPD data for THL-NIC cocrystals extruded at 500 g/h (**Top**) and 1.5 kg/h (**Bottom**), after 12 months at accelerated conditions where the measured pattern is represented with the blue line, the simulated pattern with the red line and the different pattern in grey. Refinement values displayed below.

Stability studies of the bulk substances and extruded cocrystals were undertaken at accelerated conditions (40 °C and 75% RH) for a 12-month period. DSC analysis showed only a single cocrystal melting point, indicating excellent thermal stability, with the absence of any recrystallized product [65,66]. Further XRPD analysis (Figure 11) provided near identical diffraction peaks with only a small amount of THL recrystallizing under accelerated conditions, with 1.4% present in the 500 g/h batch and 1.8% recrystallized in the 1.5 kg/h batch. This is likely due to NIC present in the cocrystal forming hydrogen bond with the water molecules high humidity conditions, causing the THL component to begin to dissociate in the absence of the NIC. It has been shown that water molecules as H-bond donors exhibit a propensity to interact with the amide group of NIC to form a different synthon to those present in THL-NIC cocrystals [67]. This type of cocrystal disassociation is common at humidities of 75% where there are large solubility differences between the API and coformer [68]. As the NIC interacts with the water molecules at high humidities, it will cause a partial disassociation of the cocrystal, through the recrystallization of the unbound THL [69,70]. By comparing this data to that of the bulk THL shown in Figure 7, it can be seen that a peak characteristic of bulk THL has appeared at 12.5 2θ.

## 4. Conclusions

HME processing was used for the scale-up of the manufacturing of pharmaceutical THL-NIC cocrystals. The scale-up of high purity THL-NIC cocrystals was seen to be dependent on keeping a consistent residence time through alteration of screw speed based on the feed rate. This is in contrast to previously published works from our group which showed temperature as an important factor to be changed when scaling up cocrystal production. During manufacture, one should strive to change as few processing parameters as possible, to avoid any unwanted side effects. By changing only the screw speed and feed rate in scaled-up batches to match the residence time, one can prevent the manufacture of partially cocrystallized batches and degraded products, leading to poor crystallinity. The formed cocrystals were processed below their eutectic temperature while the screw speed had to be adjusted during the scale up process to compensate for the increased feed rate. By using theophylline as a model drug, this study has shown that the scale-up in production of pharmaceutical cocrystals can be easily achieved through HME, without sacrificing cocrystal purity, solubility and with minimal editing of process parameters.

## Figures and Tables

**Table 1:** Scale-up trials of theophylline–nicotinamide (THL-NIC) cocrystals by investigating the effect of critical process parameters.

No	Temperature (Max) (°C)	Screw Speed (rpm)	Throughput (Kg/h)	Cocrystals
F1	145	70	0.5	X
F2	145	100	0.5	X
F3	165	70	0.5	✓
F4	165	85	0.5	X
F5	165	70	1.5	X
F6	165	100	1.5	✓
F7	185	70	0.5	X
F8	185	100	0.5	X

## Data Availability

The data presented in this study are available on request from the corresponding author.

## Supplementary Materials

The following are available online at https://www.mdpi.com/1999-4923/13/3/419/s1, Table S1: Table detailing the temperature parameters for each individual heating zone for the optimization trials.
