# Supplementary material for: Continuous Manufacture and Scale-Up of Theophylline-Nicotinamide Cocrystals
Source: Pharmaceutics. 2021 Mar 20;13(3):419. doi: 10.3390/pharmaceutics13030419 (PMC8004052; doi:10.3390/pharmaceutics13030419)
Supplement: Supplementary file 1 [file pharmaceutics-13-00419-s001.pdf]

pharmaceutics

MDPI

Article

Supplmentary Materials: Continuous manufacture and scale – up of theophylline – nicotinamide cocrystals

Steven A. Ross, Andrew Hurt [1], Milan Antonijevic, Nicolaos Bouropoulos, Adam D. Ward, Pat Basford, Mark McAllister, and Dennis Douroumis

Figure S1. Table detailing the temperature parameters for each individual heating zone for the optimization trials.

Exp No	Heat zone 1 (◦C)	Heat zone 2 (◦C)	Heat zone 3 (◦C)	Heat zone 4 (◦C)	Heat zone 5 (◦C)	Heat zone 6 (◦C)	Heat zone 7 (◦C)	Heat zone 8 (◦C)	Heat zone 9 (◦C)
**F1**	50	110	145	145	145	145	145	145	145
**F2**	50	110	145	145	145	145	145	145	145
**F3**	50	110	145	165	165	165	165	165	165
**F4**	50	110	145	165	165	165	165	165	165
**F5**	50	110	145	165	165	165	165	165	165
**F6**	50	110	145	165	165	165	165	165	165
**F7**	50	110	145	185	185	185	185	185	185
**F8**	50	110	145	185	185	185	185	185	185
